# Through the lens of the clinician: autopsy services and utilization in a large teaching hospital in Ghana

**DOI:** 10.1186/1756-0500-7-943

**Published:** 2014-12-23

**Authors:** Alfred E Yawson, Edem Tette, Yao Tettey

**Affiliations:** Department of Community Health, School of Public Health, College of Health Sciences, University of Ghana, Room 46, P. O. Box 4236, Korle-Bu, Accra, Ghana; Department of Pathology, University of Ghana School of Medicine and Dentistry, College of Health Sciences, Korle-Bu, Accra, Ghana; Office of the Provost, College of Health Sciences, University of Ghana, Korle-Bu, Accra, Ghana

**Keywords:** Autopsy service, Barriers to autopsies, Clinicians, Teaching hospital, Ghana

## Abstract

**Background:**

Declining hospital autopsy rates in many countries have generated considerable concern. The survey determined challenges of the autopsy service in a large Teaching Hospital in Ghana, from the perspective of clinicians.

**Methods:**

This was a cross-sectional study of doctors at the Korle-Bu Teaching Hospital (KBTH) over in 2012. The data was collected using a 69 item self-administered structured questionnaire. In all a total of 215 questionnaires were sent out and 119 doctors responded. Data was collected on the challenges of the autopsy services and barriers to autopsy request from the perspectives of clinicians. Survey data were analyzed by simple descriptive statistics (i.e. proportions, ratios and percentages. Data from survey was analyzed with SPSS version 21.

**Results:**

The most common reasons for requesting autopsies were to answer clinical questions, 55 (46.2%) and in cases of uncertain diagnosis, 54 (45.4%). Main demand side barriers to the use of autopsy services by clinicians were reluctance of family to give consent for autopsy 100 (84%), due to cultural and religious objections 89 (74.8%), extra funeral cost to family53 (44.5%) and increased duration of stay of body in the morgue 19 (16%). Health system barriers included delayed feedback from autopsy service 54 (45.4%), difficulties following up the autopsy process 40 (33.6%) due to uncertainties in the timing of particular events in the autopsy process, and long waiting time for autopsy reports 81 (68.1%). More than a third of clinicians 43 (36.2%), received full autopsy report beyond three weeks and 75 (63.1%) clinicians had concerns with the validity of reports issued by the autopsy service (i.e. reports lack specificity or at variance with clinical diagnosis, no toxicological, histological or tissue diagnoses are performed).

**Conclusion:**

The autopsy service should restructure itself efficiently and management should support the provision of histological and toxicological services. Strengthening internal and external quality improvement and control of autopsies in the Hospital are essential.

## Background

Declining hospital autopsy rates in many countries have generated considerable concern among sections of the medical profession [1, 2]. Clinicians are primarily responsible for initiating hospital autopsy requests, and knowledge on their views, attitudes and perceptions of health institutional challenges to requesting and obtaining autopsy reports are critical [3–5]. Demand side and supply side (health system) factors influence autopsy rates in hospitals [5, 6]. Demand side factors include attitudes and behaviours of patients and relations of patients, and sociocultural and religious factors. Supply side factors include confidence in clinical diagnosis, use of sophisticated medical diagnostic technology, fears of legal suits by requesting clinicians, and inadequate material and human resources [1, 5–10].

It is essential to determine the challenges to efficient delivery of autopsy service and barriers to utilization of the service in the Korle-Bu Teaching Hospital, the largest tertiary health care delivery facility in Ghana [10, 11]. Such an evaluation from the clinicians who utilize autopsy services is vital for indicating where resources should most effectively be allocated [1, 12, 13]. A potential source of data on patterns of mortality for health policy and planning is data from hospitals; hospital records and autopsy data provide this useful data for planning and resource allocation [10, 12–15].

Clinicians’ general interest in autopsy has been shown to be a dominant factor influencing intention to request autopsy and shown among clinicians in the Teaching Hospital in Ghana [1, 5, 10]. However, health system factors including adequacy of personnel, ability to provide histological and toxicological services to support and confirm clinical diagnosis may be important barriers to clinicians’ request of autopsies. It is expensive to run an effective clinical pathology service in a low income setting [7, 16].

There is a global shortage of trained pathologists and insufficient capacity to cover surgical autopsy as well as biopsy, especially in areas facing extreme resource constraints. In most African countries, the health services are overwhelmed with the increasing mortality from the devastating effects of HIV which increases the workload on the already inadequate staff [11, 17, 18]. Efficient autopsy service depends on systems organization and adequate personnel [18]. The autopsy service currently has only 6 consultant pathologists, 6 specialist pathologists and 5 residents in training [10, 11].

The goal of this survey was to determine the views of clinicians on the autopsy services in this large tertiary health facility in Ghana. Clinicians are primarily responsible for initiating the autopsy process in the Hospital, their identification of barriers and challenges hold great value in implementing measures to improve autopsy services, utilization of autopsies and patient care in the Teaching Hospital.

## Methods

This was a cross-sectional study of doctors at the Korle-Bu Teaching Hospital over in 2012. The data was collected using a 69 item self-administered structured questionnaire.

### Site of study

The Korle-Bu Teaching Hospital (KBTH), a tertiary health care facility in Ghana, was the survey site. The KBTH has a bed capacity of 2000 and over 3000 staff [7]. In 2010, 29,757 clients were seen on the average per month, average daily outpatient attendance was 1500 and average daily admission was 150 and over 4000 mortality from the clinical departments [11]. Autopsy service in KBTH is provided by the Department of Pathology. The Department of Pathology is a nucleus of the University of Ghana Medical School (UGMS) provides surgical pathology, cytology/cytopathology and autopsy services. Due to challenges with logistics and equipment, surgical autopsies in the department seldom include histological services as demonstrated in a recent study in the Teaching Hospital [10, 11]. It is involved in undergraduate, postgraduate and residency training and research in surgical pathology, cytopathology and autopsy. The department manages and operates the mortuary unit which has a storage capacity of 350 bodies and stores between 8,500 and 10,000 bodies annually. The mortuary serves the Hospital and the general public [10, 11].

Overall between 3,000 and 5,500 (average 4,100) autopsies are performed annually from within and outside the KBTH; about 20% of these are hospital autopsies. In the Hospital the autopsy rates are as follows; Department of Child Health (average 30%), Department of Obstetrics and Gynaecology (average 30%), Department of Surgery (average 38%) and Department of Medicine (average 60%) [10, 11]. Histological services are provided by limited and therefore not all autopsy reports include histological diagnosis [10]. There are no private facilities which offer surgical autopsy services and the teaching hospital handles all the hospital cases and referrals from other health institutions within Accra and outside Accra [11].

### Sampling methods/selection of survey sites

Doctors from the main Clinical Departments i.e. Internal Medicine, Surgery and Allied Surgery, Child Health and Obstetrics and Gynaecology (OBGYN) were involved in the survey. These service centres were selected based on the clinical services they provide and the critical importance of autopsies in the practice of these doctors. The survey was a total enumeration of all doctors in these departments. The questionnaires were distributed in each department through the heads of department.

### Study population

The population for the survey were doctors of all categories in the Hospital. These included house officers, medical officers, senior medical officer/residents, senior residents and consultants in all the clinical departments of the Korle-Bu Teaching Hospital.

### Data collection

The questionnaire solicited information on the background of the respondent including sex, current status and Department of work as well as the main reasons for autopsy request. Background data whether doctors attend departmental clinico-pathological or mortality meetings and whether pathologists in the Hospital are involved in these mortality meetings. Data was also collected on the barriers and challenges on the request and use of autopsies by doctors and the challenges to autopsy service provision in the Hospital.

### Data analysis

Survey data were analyzed by simple descriptive statistics (i.e. proportions, ratios and percentages). Data were summarized in tables. Data from survey were entered into Microsoft Excel 2007 and imported into SPSS version 21, and analyzed.

### Ethical issues

Ethical Approval for the survey was obtained from the University of Ghana Medical School Ethical and Protocol Review Committee (Protocol Identification Number: MS-Et/M.11-P 5.8/2011–2012 ). Clearance was also received from the Management of the Korle-Bu Teaching Hospital and Heads of Clinical units where survey was conducted.

## Results

A total of 215 questionnaires were sent out and 119 clinicians responded, giving a response rate of 55.3%. There were more males 67 (56.3%), than females 52 (43.7%); with a male: female ratio of 1.3: 1. The sex distribution of respondents were not different from all 215 clinicians who had questionnaire sent to (in the 215 clinicians 125 (58%) were males). Majority of the clinicians involved in the survey were senior medical officers/residents, 45 (37.8%). There were consultant, 19 (16%) involved in the survey, as indicated in Table 1.

**Table 1:** **Status and sex distribution of doctors involved in survey in the Teaching Hospital**

Current position	Sex of respondent		Total
	Male	Female	
House officer	18 (29.6)	16 (30.8)	34 (28.6)
Medical officer	1 (1.5)	3 (5.8)	4 (3.4)
Senior medical officer/resident	28 (41.8)	17(32.7)	45 (37.8)
Senior resident	10 (14.9)	7 (13.5)	17 (14.4)
Consultant	10 (14.9)	9 (17.3)	19 (16.0)
**Total**	**67 (100)**	**52 (100)**	**119 (100)**

Overall, 92 (77.3%) of clinicians did request for autopsy in the previous six months and the two most common reason for requesting autopsies were to answer clinical questions, 55 (46.2%) and in cases of uncertain diagnosis, 54 (45.4%) as shown in Table 2. Of the 92 (77.3%) clinicians who requested for autopsy in the previous six months only 23 (19.3%) clinicians had personal interaction with the pathologist during autopsy process. A majority of clinicians, 88 (73.9%) had not attended any autopsy demonstrations in the past 6 months however, almost all clinicians 111 (93.3%) attended monthly mortality or clinic-pathological meeting in their Department/unit where autopsy reports discussed. Attendance of a pathologist at such meetings was very low.

**Table 2:** **Background characteristics of clinicians involved in the Teaching Hospital survey**

Characteristic	Frequency	Percentage
**Reasons for autopsy request**		
Answer clinical questions	55	46.2
Uncertain diagnosis	54	45.4
Confirm clinical diagnosis	40	33.6
Coroners case	39	32.8
Family request	2	1.7
Explain sudden questionable deaths	1	0.8
Total	119	100.0
Number of clinicians who requested for autopsy in previous six moths	92	77.3
Total	119	100.0
**Personal interaction with pathologist during the process of autopsy**		
Yes	23	19.3
Total	119	100
**Number of autopsy demonstrations attended by doctors in past 6 months**		
none	88	73.9
1	12	10.1
2	3	2.5
3 or more	7	5.9
Total	119	100
**Monthly mortality or clinic-pathological meeting in Department/unit**		
Yes	111	93.3
Total	119	100
**Autopsy reports discussed at meetings**		
Yes	90	75.6
Total	119	100
**Pathologist or personnel from Department of Pathology participate in mortality meetings**		
Yes	3	2.5
Total	119	100.0

The two main demand side barriers to autopsy request from the perspectives of clinicians as indicated in Table 3, were reluctance of family to give consent for autopsy to be performed (especially on patients advanced in age) 100 (84%), as well as due to cultural and religious objections raised by families 89 (74.8%). Other factors included perceived increased or extra funeral cost for the family and increased duration of stay of body in the morgue due to the autopsy process. The two main supply side or health system barriers were delays in obtaining feedback from the autopsy service 54 (45.4%) and difficulties following up the autopsy process (due to uncertainties in the timing of particular events in the autopsy process), 40 (33.6%). Other health system barriers identified were limited ability of requesting doctors to use autopsy information to improve patient care, and lack of knowledge and circumstance under which autopsy is permitted.

**Table 3:** **Clinicians views on general factors hindering autopsy services in the Teaching Hospital**

Main Barrier to autopsy request	Frequency	Percentage
Reluctance of family to give consent	100	84.0
Cultural and religious objections (Muslims and traditional royal persons)	89	74.8
Delay in feedback from the pathological service	54	45.4
Increased cost to family	53	44.5
Difficulty following up	40	33.6
Age at death	29	24.4
Lack of feedback from the pathological service	28	23.5
Increased duration of stay of body in morgue	19	16.0
Busy clinical service	11	9.2
Limited ability to use autopsy information to improve care	10	8.4
Lack of knowledge/circumstance under which autopsy is permitted	6	5.0
Unintended consequences	4	3.4
Others (Fears of being sued if diagnosis was missed, Request form too complicated, Missing folders/health records)	8	6.7
**Total**	**119**	100.0

As indicated in Table 4, clinicians deemed the main health system barriers to autopsy service in the Teaching Hospital to be the long waiting time for autopsy reports 81 (68.1%). The mean time taken for receipt of full autopsy report was beyond three weeks, for over a third of clinicians 43 (36.2%). In all 75 (63.1%) clinicians had concerns with the validity of reports issued by the autopsy service. The specific concerns of the clinicians were that pathological report received were sometimes at variance with the clinical diagnosis, 16 (13.4%) and that reports do not state specific findings 6 (5%). In addition, neither toxicological nor histological nor tissue diagnoses were done; hence clinical diagnosis is reported without histological diagnosis.

**Table 4:** **Clinicians views on Health system challenges to autopsy services in the Teaching Hospital**

Characteristic	Frequency	Percentage
**Average time taken for receipt of full autopsy report**		
<1 week	14	11.8
1-2 weeks	42	35.3
3-4 weeks	34	28.6
1-3 months	7	5.9
>6 months	2	1.7
Total	119	100
**Waiting time for autopsy report unduly long**		
Yes	81	68.1
Total	119	100
**Concerns about validity of reports issued by autopsy service**		
Not at all	33	27.7
Just a little	46	38.7
Often	15	12.6
Unable to judge	14	11.8
Total	119	100
**Specific concerns on the autopsy services**		
Pathological report at variance with clinical diagnosis	16	13.4
Specific findings not stated, too generalized	6	5.0
Autopsy findings are no different from clinical diagnosis	5	4.2
No toxicology reporting	4	3.4
No histology reports received	4	3.4
Others (Clinical diagnosis given instead of histological diagnosis, clinical queries not addressed, provides no added information)	5	4.2
Total	119	100

Despite these concerns, 72 (60.5%) of the clinicians patronized the Teaching Hospital’s autopsy service.

## Discussion

Autopsy reports provide useful guidance to clinical management of patient and assist in the provision of precise information on the cause of death of patients [19, 20]. Despite the well established role of autopsy in disclosing clinical diagnostic inaccuracy among clinicians, autopsy rates have been declining gradually over several decades in many parts of the World [9, 21, 22].

In this study, clinicians of all categories in the Hospital were seen to request autopsies and the main reasons for requesting autopsies were to answer clinical questions and in cases of uncertain diagnosis. These reasons are in conformity with other studies which indicated that autopsies provide a good index of the quality of patient care, in terms of the accuracy of clinical diagnosis and the quality of treatment given [2, 7, 23].

Clinicians initiate the autopsy process in the Hospital, identification of major barriers within and outside the health system by this group of health workers can provide useful lessons in implementing measures to optimize service delivery [5]. Lack of cooperation and reluctance of family to give consent for autopsy to be performed especially on patients who are advanced in age hinder the optimum use of autopsy services. Indeed families may be apprehensive on the autopsy process due to cultural and religious objections where bodies must be buried soon after death or the fear of extra funeral cost due to increased duration of stay of body in the morgue due to the autopsy process. These barriers concurred with factors suggested elsewhere as possibly having some effect on autopsy requests [1, 24]. Providing more structured, organized and timely autopsy services, fast-tracking processes if religion is a barrier, and effective communication with families and relations of patients may be effective. The Hospital should conduct regular patient and public education on the utility and benefit of autopsies, provide each department/unit of the Hospital clear guidelines on autopsies and create positive attitudes toward autopsies among doctors/residents in training. The residents, who most often deal directly with families/relations need to be equipped with the skills to effectively communicate the need for autopsies.

The expenses involved in autopsies as grounds for reluctance of families to consent to clinician’s request is critical especially in low income settings [7, 16]. Families who have a catastrophic health event spend valuable limited family resources on caring for the sick relative. The last thing they will entertain is additional cost of autopsy on the loved one who has already passed. To improve utility of autopsies to clinical care and mortality data in this large teaching hospital and others in the country, national policies included cost of targeted autopsies under the national health insurance scheme may be worth considering.

Major health system bottlenecks to autopsy services identified were inadequate skilled personnel and lack of equipment to provide timely surgical autopsies and other specific services such as cytology/cytopathology; currently most autopsies in the Hospital are limited to surgical pathology [10]. Inability to provide histological and toxicological support is mainly as a results of limited human and technological resources-materials and equipment [11]. It is the belief of authors that, ability of the service to provide histological and toxicological services would improve the confidence reposed in the reports generated from the autopsy service and provide additional information for requesting clinicians. In Ghana human and basic resource for pathological services pose huge challenges at all levels of health care delivery (national, regional and district). These critical issues of improving human and basic resource for the autopsy service to garner effective service delivery, deserve utmost attention by hospital managements and national governments in low income settings.

Under the current difficult circumstances under which the autopsy service operates, (especially regarding delays in providing autopsy reports to clinicians), certain restructuring of processes in the service may be efficient. The service should designate residents doctors in the department of Pathology to specific department/units of the hospital to be involved in their clinico-pathological meetings and to deal promptly with their autopsy request as well. The assigned responsibility may improve communication of pathologist and clinicians; the current order of services probably hinder effective engagement of pathologists and clinicians.

Management of autopsy service and Hospital Management should institute internal and external quality improvement and control measures to regulate and support autopsies in the Hospital. Regular operational and hospital based research and reviews (satisfaction surveys among clients, clinicians and pathologists) could identify bottlenecks to optimum service provision to guide implementation of corrective measures. The role, duties and responsibilities of requesting clinicians and pathologists in the Teaching Hospital should be critically examined to bridge gaps in service delivery.

Human resource challenges have been seen as one of the major factors contributing to the decline of autopsies in most parts of the world, especially in resource limited settings [7, 10, 16].

The ultimate need to increase human capital for the service cannot be ignored and Hospital Management and the Ministry of Health, should see training of more pathologists as a priority.

The fear of legal suits due to diagnostic inaccuracies and inadequate knowledge on criteria for reportable deaths (coroners case) have been demonstrated in other studies among clinicians [1, 7, 8, 25]. This conforms to findings from this Teaching Hospital based survey. Institutional provision of a structured capacity building programme on autopsy requests, death certification, coronial system and communication skills will garner more confident clinicians [6, 10]. An initiative to achieve this could be a structured continuous medical education programme organized as collaboration between institutional care division of Ministry of Health/Ghana Health Service and the autopsy service in the Teaching Hospital. This should provide the basis to improve medico-legal education of all clinicians on regular basis in the Hospital [6, 25]. Implementing such an initiative may reduce the apprehension of clinicians - fear of being sued if diagnosis was missed, features of the coronial system which are poorly understood by clinicians and autopsies in terminally ill patients [1, 7, 8, 25]. It must be emphasized that, despite advances in medical diagnostic technology, diagnostic inaccuracies do occur even in developed settings; thus autopsy requests are imperative resource limited settings [11].

### Limitations

A limitation of this analysis was the relatively low response rate of 55.3%. Clinicians who did not respond probably might have provided other dimensions to the theme of the survey. However, all categories of clinicians were involved and non response did not cluster in any particular category of clinicians.

## Conclusion

All categories of clinicians from all clinical departments value autopsy reports as essential in improving clinical practice and patients care. However, majors challenges confront the provision of efficient autopsy service in the Hospital. Critical demand and health system factors need to be improved for optimum autopsy service provision and utilization in this large tertiary health care facility.

## Authors’ information

Y Tettey is a Professor of Pathology of the Pathology Department, University of Ghana Medical School and current Provost of the College of Health Sciences, University of Ghana, Accra.

E Tette, is a consultant paediatrician and public health physician and current head of the Department of Community Health, School of Public Health, College of Health Sciences, University of Ghana, Korle-Bu, Accra.

AE Yawson is a consultant public health physician and lecturer in the Department of Community Health, School of Public Health, College of Health Sciences, University of Ghana, Korle-Bu, Accra.
